# Decreased Empathic Responses to the ‘Lucky Guy’ in Love: The Effect of Intrasexual Competition

**DOI:** 10.3389/fpsyg.2016.00660

**Published:** 2016-05-11

**Authors:** Li Zheng, Fangxiao Zhang, Chunli Wei, Jialin Xu, Qianfeng Wang, Lei Zhu, Ian D. Roberts, Xiuyan Guo

**Affiliations:** ^1^Key Laboratory of Brain Functional Genomics, Ministry of Education, Shanghai Key Laboratory of Brain Functional Genomics, School of Psychology and Cognitive Science, East China Normal UniversityShanghai, China; ^2^School of Psychology and Cognitive Science, East China Normal UniversityShanghai, China; ^3^Shanghai Key Laboratory of Magnetic Resonance and Department of Physics, East China Normal UniversityShanghai, China; ^4^Department of Psychology, Fudan UniversityShanghai, China; ^5^Department of Psychology, The Ohio State UniversityColumbus, OH, USA; ^6^Shanghai Key Laboratory of Magnetic Resonance and School of Psychology and Cognitive Science, East China Normal UniversityShanghai, China

**Keywords:** pain empathy, intrasexual competition, fMRI, AI, aMCC, MPFC

## Abstract

People have a greater desire to date highly attractive partners, which induces intrasexual competition between same-sex individuals. The present study used functional magnetic resonance imaging to explore whether and how intrasexual competition modulates pain empathy for a same-sex rival and the underlying neural mechanism. Participants were scanned while processing the pain of a same-sex ‘lucky guy’ who had an attractive partner and one with a plain partner. The results revealed that participants reported lower pain intensity for the lucky guy. Neurally, reduced pain-related activations in anterior insula and anterior mid-cingulate cortex and increased activations in right superior frontal gyrus (SFG) and medial prefrontal gyrus (MPFC) were found for the lucky guy compared to the one with a plain partner. Right SFG and MPFC activations could predict participants’ subsequent pain intensity ratings for the lucky guy. These findings suggest intrasexual competition can modulate normal empathic responses.

## Introduction

Evolutionarily speaking, physical attractiveness is linked to youth, health, and female fertility (Sugiyama, 2005). Physical attractiveness also provides information about mate-relevant economic or social factors, such as income or hunting ability (Harper, 2000) and plays an important role in mate selection (Grammer et al., 2003; Gangestad and Scheyd, 2005). Both males and females have a greater desire to date highly attractive partners (Greitemeyer, 2010). The common motive to obtain and maintain access to partners, especially highly desirable partners, can often induce intrasexual competition (Buunk and Massar, 2012). Emerging evidence has demonstrated that intrasexual competition can affect people’s attitudes. That is, people tend to dislike same-sex rivals, exhibiting negative evaluations and hostile behaviors toward them (Fisher, 2004; Griskevicius et al., 2009). Previous studies have revealed that the attitudes held toward others impact empathy for their suffering and pain (Singer et al., 2006; Cheng et al., 2010). This leads to the question: are empathic responses to the same-sex rival, especially when the rival is a ‘lucky guy’ who obtains the love of a highly attractive partner, modulated by intrasexual competition? The goal of the present study was to investigate the impact of intrasexual competition on empathy and the underlying neural mechanisms.

Empathy refers to the ability to understand and experience the emotional and affective states of another person (Decety and Jackson, 2004; Singer and Lamm, 2009; Decety, 2011). Recently, a number of neuroimaging studies on pain empathy have demonstrated that the perception or imagining of others’ pain activates a pain matrix similar to what is engaged in the first-hand experience of pain (Derbyshire, 2000; Price, 2000; Jackson et al., 2006), including the bilateral anterior insula (AI), anterior cingulate cortex (ACC), and anterior mid-cingulate cortex (aMCC; e.g., Singer et al., 2004; Jackson et al., 2006; Lamm et al., 2007, 2011; Saarela et al., 2007; Fan et al., 2011; Guo et al., 2012, 2013; Zheng et al., 2016). In addition, activation in medial prefrontal cortex (MPFC) was associated with decreased empathic responses to others’ pain (Cheng et al., 2007). Converging with evidence showing the role of MPFC in cognitive inhibitory control and emotion regulation (Floden and Stuss, 2006; Banks et al., 2007; Phillips et al., 2008), activation in MPFC during empathy for others’ pain may reflect a down-regulation of empathic pain (Cheng et al., 2007).

Although, a key feature of empathy is the observer’s experience of an affective state that is isomorphic to another person’s affective state, empathy is not simply an automatic resonance of the target’s state (De Vignemont and Singer, 2006). Previous neuroimaging studies have revealed that individuals’ attitudes toward empathy targets influence empathy for their pain. Decety et al. (2010) found that, compared with healthy controls, the more participants blamed targets for contracting AIDS from illegal intravenous drug use, the less empathy participants had for the target’s pain, indicating that empathic responses were modulated by an *a priori* negative attitude toward the empathy target. In a study conducted by Singer et al. (2006), participants, especially males, showed decreased responses in brain regions associated with empathy when observing an unfair person who they disliked receiving pain. Given that intrasexual competition leads to negative attitudes toward same-sex rivals (Fisher, 2004; Griskevicius et al., 2009), we predicted that observers would show reduced behavioral and neural empathic responses to their pain.

**FIGURE 1:**
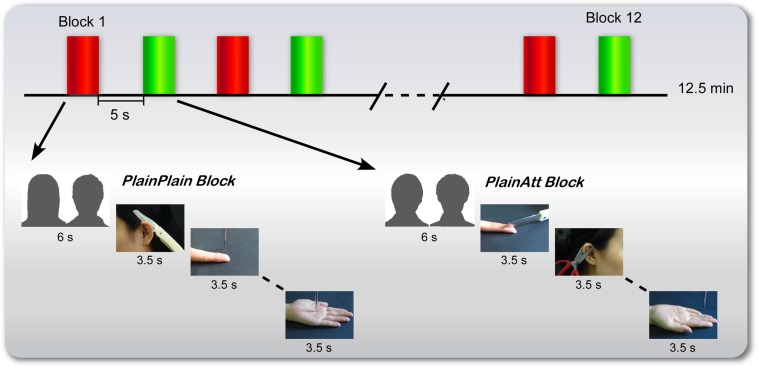
**Procedure for female participants.** There were six blocks for each plain model (red for PlainPlain and green for PlainAtt), with a 6-s cue presented before each block to indicate which plain model would be the target in the following block. The cues (here indicated by silhouettes) were pictures of two couples in the experiment. Four painful pictures and four non-painful pictures of that specific model were randomly presented with null trials, each lasting 3.5 s. The presentation order of attractive and plain model blocks was counterbalanced across participants. For male participants, the same procedure was used, with changed cues and pictures.

To test this prediction, an functional magnetic resonance imaging (fMRI) study was designed. Before the experiment, four plain models (two females and two males) and two attractive models (one female and one male) were selected. Participants were informed that one of two plain same-sex models had an attractive partner (PlainAtt), i.e., the ‘lucky guy’ in love in the experiment; another had a plain partner (PlainPlain). Then participants were scanned while viewing a series of pictures showing PlainPlain or PlainAtt (targets were always the same-sex as the participants) in painful or non-painful situations (**Figure 1**). We predicted that participants would report more negative attitudes and decreased pain intensity ratings for PlainAtt than for PlainPlain. At the neural level, decreased pain-related brain activations in AI and ACC/aMCC and increased activation in MPFC, which has been associated with the regulation of empathic pain, would be observed for PlainAtt relative to PlainPlain.

## Meterials And Methods

### Participants

Twenty right-handed participants (12 females, mean age = 21.70, *SD* = 1.89) were recruited from the university community to participate in this experiment. None of the participants had a history of neurological or psychiatric disorders. All participants received monetary compensation for their participation. All of them had normal or corrected-to-normal vision. All participants gave informed consent before scanning. The study was approved by the local ethics committee.

### Materials

A total of 192 pictures collected from two females and two males volunteers were used as stimuli. For each volunteer, 24 painful pictures, depicting their bilateral ears (not including faces), palms, and index fingers in four kinds of nociceptive situations (cutting with a knife or a pair of scissors and pricking with a needle or an awl), were paired with a corresponding non-painful situation, in which the nociceptive tool did not touch the ear, palm, or finger, but was placed next to the body part (in total, 48 pictures for each volunteer). All of the pictures were 500 × 350 pixels in size. Ninety-six pictures from the two males volunteers were used as stimuli for male participants, while ninety-six other pictures from the two females volunteers were used as stimuli for female participants.

### Procedure

Before the experiment, we collected 38 free face pictures (19 females and 19 males) from the Internet. From 19 females candidates, one attractive female model and two plain females models were selected according to the attractiveness and liking ratings of 10 males (not participants). The attractive model got the highest attractiveness and liking ratings, while the two plain models were ranked in the last quarter of the ratings. There were significant differences between the attractiveness and liking ratings of the attractive model and the plain models (*t*s > 3.46, *p*s < 0.01), but no significant attractiveness or liking rating differences between the two plain models. From 19 male candidates, one attractive male model and two plain male models were selected in the same way, with significant differences between the attractive model and plain models (*t*s > 6.20, *p*s < 0.01) and no significant differences between the two plain models. For each participant, face pictures of two couples were presented during the fMRI scan. The first couple was composed of one of the two plain same-sex models paired with the attractive opposite-sex model. The second couple was composed of the other plain same-sex model paired with one of the two plain opposite-sex models. Which plain model was paired with the attractive opposite-sex model was counterbalanced between participants.

Before scanning, participants were informed that they would view pictures of two couples and that the same-sex models from these couples would be their empathy targets. They were told they would view a series of pictures depicting the empathy target in painful or non-painful situations.

During the experiment, six blocks were set for each plain same-sex model (PlainAtt or PlainPlain; **Figure 1**). Before each block, a 6-s cue trial (i.e., a picture of one of the couples) was presented to inform the participants which plain same-sex model was the target in the following block. Each block consisted of four painful pictures and four non-painful pictures of the model indicated by the cue with null events (only fixation cross) randomly interspersed. Each trial was presented for 3.5 s followed by a jittered fixation cross from 0.5 to 1.5 s. PlainAtt and PlainPlain blocks were alternated. The presentation orders of the different blocks were counterbalanced across the participants. Each block lasted for 48.5 s, followed by a 5-s rest. The participants were asked to view the pictures attentively. The manipulation of grouping events into different blocks aimed to reduce the possible influence of participants’ cognitive loads to switch between two empathy targets on the modulation of neural empathic responses by intrasexual competition.

After scanning, participants were presented with the same stimuli that they viewed in the scanner again and asked to rate how much pain they felt in each situation (0–9 point Likert-type scale where 0 indicated no pain and 9 indicated extreme pain). Then, they were presented with the same cue pictures as in the scanner and asked to rate the attractiveness of the empathy targets who had an attractive or plain partner and how much they envied and liked the empathy targets with 1 point (not at all)-7 point (extreme) Likert-type scales.

### fMRI Image Acquisition and Analysis

Imaging was performed on a 3T Siemens Trio system (East China Normal University, Shanghai) with a standard head coil. Functional images were obtained using a gradient echo echo-planar imaging (GRE-EPI) sequence. Thirty-five transversal slices with 3 mm slice thickness and a 0.3-mm spatial gap were acquired (*TR* = 2200 ms, *TE* = 30 ms, *FOV* = 220 mm, flip angle = 90°, matrix size = 64 × 64). There was only one run of functional scanning which was about 13 min (346° EPI volumes). Before the functional imaging, a high-resolution structural image was acquired using a T1-weighted, multiplanar reconstruction sequence (MPR; 1 mm× 1 mm× 1 mm).

Participants’ data were analyzed separately using SPM8 (Statistical Parametric Mapping, Wellcome Department of Imaging Neuroscience, London, UK). During data preprocessing, the first five volumes were discarded to allow for T1 equilibration effects. The functional images were corrected for the delay in slice acquisition and were realigned to the first image to correct for interscan head movements. The individual T1-weighted, 3D structural image was co-registered to the mean EPI image generated after realignment. The co-registered structural image was then segmented into gray matter (GM), white matter (WM), and cerebrospinal fluid (CSF) using a unified segmentation algorithm (Ashburner and Friston, 2005). The functional images after slice timing and realignment procedures were spatially normalized to the Montreal Neurological Institute (MNI) space (resampled to 2 mm × 2 mm × 2 mm) using the normalization parameters estimated during unified segmentation and then spatially smoothed with a Gaussian kernel of 8 mm full-width half-maximum (FWHM).

Statistical analyses were then carried out with the general linear model (GLM) implemented in SPM8. At the first-level analysis, four types of conditions were defined based on the attractiveness of the empathy target’s partner and painful or non-painful stimuli the target received: (1) PlainAtt in the painful situations (PlainAttP); (2) PlainAtt in the non-painful situations (PlainAttN); (3) PlainPlain in the painful situations (PlainPlainP); (4) PlainPlain in the non-painful situations (PlainPlainN). All the conditions were modeled as 3.5 s long from the onset time of the pictures and convolved with a canonical hemodynamic response function (HRF). The models additionally included cues (also convolved with a canonical HRF) and six movement parameters derived from realignment as covariates of no interest. High pass temporal filtering with a cutoff of 180 s was also applied in the models. For each participant, simple main effects for each of the four conditions were computed with the ‘10’ contrast, respectively, at the first-level analysis. The resulting four first-level individual contrast images for each participant were then fed into a second-level full factorial ANOVA.

The main effect of pain was defined using the (PlainAttP + PlainPlainP) – (PlainAttN + PlainPlainN) and the reverse contrasts. The interaction between pain and partner’s attractiveness defined by the (PlainPlainP – PlainPlainN) – (PlainAttP – PlainAttN) and the reverse contrasts were also computed to explore how the empathic brain responses changed. A cluster-level threshold of *p* < 0.05 (FWE) and a voxel-level threshold of *p* < 0.001 (uncorrected) were used to define activations.

## Results

### Behavioral Results

The means and standard deviations of participants’ pain intensity, liking, attractiveness, and envy ratings were shown in Table 1. A 2 (pain: painful vs. non-painful) × 2 (partner’s attractiveness: PlainPlain vs. PlainAtt) repeated-measures ANOVA on pain intensity ratings revealed significant main effects of pain and partner’s attractiveness [*F*s(1,19) > 23.89, *p*s < 0.01]. The interaction was also significant [*F*(1,19) = 17.31, *p* < 0.01]. *Post hoc* analyses revealed significantly higher pain intensity ratings for painful situations than non-painful situations in both PlainPlain and PlainAtt conditions [*t*s(19) > 13.17, *p*s < 0.001]. Further analyses showed higher pain intensity rating difference (painful–non-painful) for PlainPlain than PlainAtt [*t*(19) = 4.16, *p =* 0.001].

**Table 1:** Mean ( ± SD) for envy, attractiveness, liking, and pain intensity ratings.

				Pain intensity	
	Envy	Attractiveness	Liking	Painful	Non-painful
PlainPlain	2.10 ± 1.07	3.35 ± 1.18	4.30 ± 1.26	7.09 ± 1.28	0.50 ± 1.16
PlainAtt	5.45 ± 1.05	3.00 ± 1.59	2.05 ± 0.94	5.38 ± 1.71	0.17 ± 0.51

Participants reported significantly less liking and higher envy [*t*(19) = 12.22, *p* < 0.01] for PlainAtt than for PlainPlain [*t*(19) = 5.41, *p* < 0.01]. No significant difference on the attractiveness ratings was found between PlainAtt and PlainPlain.

### fMRI Results

#### Main Effects

Brain regions related to painful stimuli vs. non-painful stimuli [(PlainAttP + PlainPlainP) – (PlainAttN + PlainPlainN)] were bilateral AI, aMCC, supplementary motor area, bilateral supramarginal gyrus, right inferior occipital gyrus, and cerebellum. The reverse contrast revealed significant activations in right superior occipital gyrus, right angular gyrus, left precuneus, aMCC, right middle frontal gyrus, right middle orbital gyrus, bilateral middle temporal gyrus, and left angular gyrus (**Table 2**). We did not report the main effect of partner’s attractiveness for it was of no interest in the current study.

**Table 2:** Brain regions showing a significant main effect of pain.

		Coordinates				
Regions of activation	Side	*x*	*y*	*z*	*T*-value	Volumes (mm^3^)
**Painful – Non-painful**						
Supplementary motor area	R	8	12	58	10.52	223952
Anterior insula	L	–30	22	4	9.54	
Anterior insula	R	42	10	0	9.24	
Anterior middle cingulate cortex	L	–6	14	42	9.07	
Supramarginal gyrus	L	–62	–22	34	9.36	30040
Supramarginal gyrus	R	68	–24	38	7.11	13400
Cerebelum	R	18	–70	–22	6.52	9136
Inferior occipital gyrus	R	32	–94	–8	5.88	1648
**Non-painful**–**Painful**						
Superior occipital gyrus	R	18	–88	20	6.98	19896
Angular gyrus	R	46	–60	28	6.86	13272
Precuneus	L	–10	–56	20	5.31	10624
Middle frontal gyrus	R	26	28	44	7.30	8848
Middle orbital gyrus	R	8	42	–12	4.87	4752
Middle temporal gyrus	R	62	–4	–18	4.87	3320
Angular gyrus	L	–46	–78	30	4.69	2760
Middle temporal gyrus	L	62	–4	–18	4.71	2360

*Coordinates (mm) are in Montreal Neurological Institute (MNI) space. L = left hemisphere; R = right hemisphere. Cluster-level, p < 0.05, FWE corrected; voxel-level, p < 0.001, uncorrected.*

#### Interactions

**FIGURE 2:**
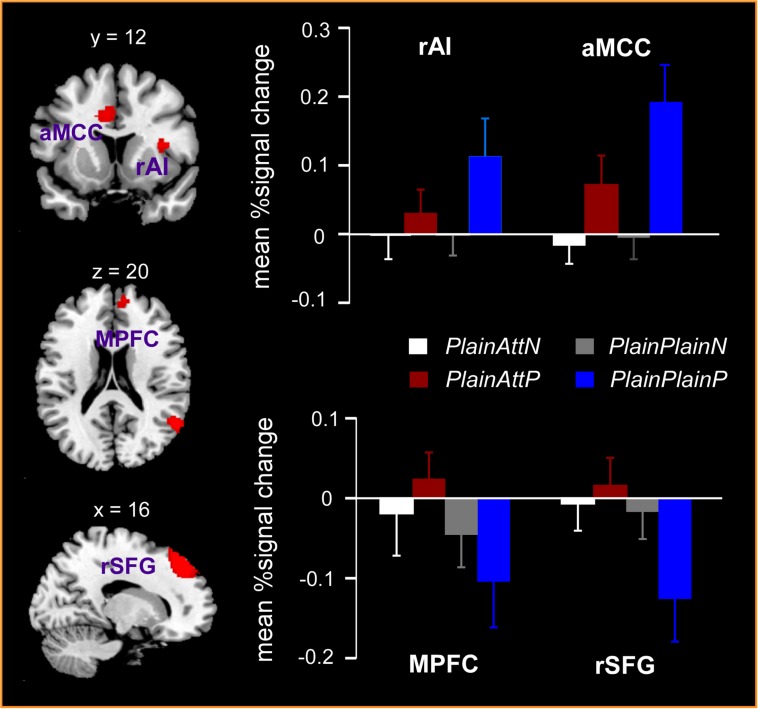
**Significant activations in the interaction between pain and the partner’s attractiveness.** Right anterior insula (AI) and anterior mid-cingulate cortex (aMCC) responded more strongly to painful simulations applied to PlainPlain than PlainAtt, whereas stronger activations in medial prefrontal cortex (MPFC) and right superior frontal gyrus (SFG) were observed for PlainAtt’s pain than for PlainPlain’s pain. No activation difference was found between PlainAtt and PlainPlain in any brain region when responding to non-painful simulations. L = left hemisphere; R = right hemisphere. Error bars indicate standard error of the mean. Cluster-level, *p* < 0.05, FWE corrected; voxel-level, *p* < 0.001, uncorrected.

The interaction between pain and partner’s attractiveness identified by the (PlainPlainP – PlainPlainN) – (PlainAttP – PlainAttN) contrast revealed significant activations in right AI, aMCC, left thalamus, left precuneus, and left precentral gyrus, while the reverse contrast revealed significant activations in MPFC, right superior frontal gyrus (SFG) and right angular gyrus (**Table 3**). Percent signal changes were extracted from all the significant voxels in the 6 mm-radius spherical regions centered on the peak or local maximum coordinates identified in the interactions (coordinates can be found in **Table 3**). Further *post hoc* analyses revealed that, when watching painful pictures, greater percent signal changes in aMCC, right AI, left thalamus, left precuneus, and left precentral gyrus were detected for PlainPlain than for PlainAtt [*t*s(19) > 3.48, *p*s < 0.01], whereas in MPFC, right SFG and right angular gyrus, greater percent signal changes was observed for PlainAtt than for PlainPlain [*t*s(19) > 5.98, *p*s < 0.01; **Figure 2**]. When watching non-painful pictures, no significant difference was found between PlainAtt and PlainPlain in any brain regions (**Figure 2**). Interestingly, for both MPFC and right SFG, we found that the difference in percent signal change between PlainAttP condition and PlainAttN condition was negatively correlated with the corresponding pain intensity rating difference (MPFC: *r* = –0.56, right SFG: *r* = –0.52, both *p* < 0.05), whereas similar analyses found no significant correlation for PlainPlain trials.

**Table 3:** Brain regions showing significant interactions.

		Coordinates				Volumes (mm^3^)
Regions of activation	Side	*x*	*y*	*z*	*T*-value	
**(PlainPlainP – PlainPlainN) – (PlainAttP – PlainAttN)**						
Precentral gyrus	L	–36	–12	44	4.16	7760
Anterior middle cingulate cortex	L	–6	10	34	4.13	
Thalamus	L	–10	–26	16	4.72	5288
Precuneus	L	–12	–54	54	5.08	4960
Anterior insula	R	36	12	14	4.09	2504
**(PlainAttP – PlainAttN) – (PlainPlainP – PlainPlainN)**						
Angular gyrus	R	54	–58	34	6.09	8056
Superior frontal gyrus	R	16	36	46	5.53	5760
Medial prefrontal cortex	R	12	54	20	3.77	

*Coordinates (mm) are in MNI space. L = left hemisphere; R = right hemisphere. Cluster-level, p < 0.05, FWE corrected; voxel-level, p < 0.001, uncorrected.*

## Discussion

The present study investigated how the empathic responses were modulated by intrasexual competition. Results showed that, at the behavioral level, participants reported less liking, lower empathic pain intensity, and higher envy for PlainAtt, the lucky guy with an attractive partner, than PlainPlain, the one with a plain partner. At the neural level, increased activations in AI and aMCC were observed in the painful situations relative to the non-painful situations. More importantly, decreased pain-related AI and aMCC activations and stronger MPFC and right SFG activations were found for PlainAtt than for PlainPlain, indicating behavioral and neural empathic responses to others’ pain were modulated by intrasexual competition.

Anterior insula and ACC/aMCC have been considered to be involved in encoding the affective dimension of first-hand pain and to play a key role in empathy for others’ pain (Singer et al., 2004). In the present study, AI and aMCC responded more strongly to the painful relative to the non-painful stimuli, which was consistent with the findings of previous studies that these areas were critically involved in empathic pain processing (Fan et al., 2011; Lamm et al., 2011). Furthermore, it has been shown that empathic neural responses in AI and aMCC are modulated by many situational factors (e.g., Xu et al., 2009; Guo et al., 2012, 2013; Feng et al., 2016). For example, a recent study found that social hierarchies established based on incidental skill in a perceptual task affected AI and aMCC activations during empathy for pain. Specifically, empathic brain activations in AI and aMCC were reduced when responding to the pain of superior-status targets relative to that of inferior-status targets (Feng et al., 2016). Interestingly, our data revealed that AI and aMCC were also involved in the modulation of pain empathy by intrasexual competition. Compared with the guy with a plain partner, when participants saw the lucky guy suffering pain, reduced AI and aMCC activations and lower pain intensity ratings were observed, indicating that the normal empathic response for the lucky guy was suppressed. It should be noted that, accompanying the decrease of empathic responses, participants reported more negative attitudes (i.e., less liking and higher envy) toward the lucky guy, suggesting that intrasexual competition altered their attitudes, which might have affected the empathy for others’ pain. This converging evidence may indicate that AI and aMCC activities are not just responsible for automatic bottom–up processing of others’ pain, but are also sensitive to the top–down regulation of pain empathy by various situational factors, such as intrasexual competition and social hierarchies.

Interestingly, the present study also found greater activation in MPFC for PlainAtt than for PlainPlain. Previous studies have demonstrated that MPFC plays an important role in cognitive inhibitory control and emotion regulation (Floden and Stuss, 2006; Banks et al., 2007; Phillips et al., 2008). Recent brain imaging studies also revealed the engagement of MPFC in the empathy for others’ pain (Cheng et al., 2007; Rameson et al., 2012). Cheng et al. (2007) observed increased MPFC activation and also negative functional connectivity between MPFC and AI when physicians who practice acupuncture (versus matched controls) viewed painful stimuli, which was interpreted as reflecting cognitive inhibition of the affective processing in the pain matrix, revealing that MPFC was related to the down-regulation of empathy for pain. The data of the present study revealed greater MPFC activation for PlainAtt than for PlainPlain during the painful (relative to non-painful) situations. Further evidence from correlation analyses showed that when participants saw the lucky guy in pain, stronger activation in MPFC was observed and lower pain intensity ratings were reported, providing support for the argument that MPFC was associated with the evaluation of others’ physical pain. Additionally, we also found that MPFC activation in the present study was accompanied by activity in right SFG. In addition to MPFC activity, Cheng et al. (2007) also observed increased right SFG activation and negative functional connectivity between right SFG and AI when physicians (versus controls) viewed painful stimuli. In the present study, right SFG was more active while processing the painful stimuli for the lucky guy and its activation predicted participants’ pain intensity ratings for the lucky guy. This evidence suggests that right SFG may have a similar function to MPFC in evaluating others’ physical pain.

The present study used fMRI to explore pain empathy for the lucky guy in love. The results revealed that individuals had more negative attitudes and reported lower pain intensity for the lucky guy with an attractive partner compared to the one with a plain partner. Neurally, reduced responses in AI and aMCC and increased activations in MPFC and right SFG were observed for the pain of the lucky guy compared to the one with a plain partner. These findings indicate intrasexual competition can affect attitudes toward same-sex rivals and modulate normal empathic responses to their suffering.

## Author Contributions

XG conceived of the project; XG, Li Zheng, and Lei Zhu designed the experiments; Li Zheng programmed the experimental scenario; Li Zheng and QW performed the experiments; JX analyzed the data with the help of Li Zheng; FZ and CW wrote the paper; all authors read and approved the manuscript. Li Zheng and FZ contributed equally to this work.

## Conflict of Interest Statement

The authors declare that the research was conducted in the absence of any commercial or financial relationships that could be construed as a potential conflict of interest.
